# Coronary ostial stenosis detected by transesophageal echocardiography after aortic valve replacement: a case report

**DOI:** 10.1186/s40981-017-0083-8

**Published:** 2017-04-11

**Authors:** Naomi Ono, Toshiyuki Sawai, Hisanari Ishii

**Affiliations:** 1 Department of Anesthesia, Tenri Hospital, 200 Mishima-cho, Tenri, Nara 632-8552 Japan; 2 Department of Anesthesiology, Osaka Medical College, Osaka, Japan

**Keywords:** Coronary ostial stenosis, Transesophageal echocardiography, Aortic valve replacement, Coronary artery bypass grafting

## Abstract

**Background:**

Coronary ostial stenosis is a life-threatening complication of aortic valve replacement (AVR). Clinical symptoms usually appear within the first 6 months after AVR (Funada and Mizuno et al., Circ J 70:1312–7, 2006), and perioperative onset is very rare.

**Case presentation:**

An 80-year-old woman with severe aortic stenosis was scheduled to undergo AVR. AVR using cardiopulmonary bypass (CPB) was successfully carried out. However, 5 min following AVR, signs of left heart failure appeared, and transesophageal echocardiography (TEE) revealed severe hypokinetic left ventricular wall motion. Left coronary ostial stenosis was diagnosed by TEE, and CPB was immediately resumed and coronary artery bypass grafting (CABG) to the left anterior descending branch was performed.

**Conclusions:**

When circulatory failure presents in the acute phase following AVR, onset of coronary ostial stenosis should be considered.

## Background

Coronary ostial stenosis is a rare but life-threatening complication of aortic valve replacement (AVR). Its process of development differs from that of arteriosclerosis, and clinical symptoms typically appear within the first 6 months following AVR [1]. Perioperative onset of the condition is very rare. Here, we report a case of sudden onset left coronary ostial stenosis during AVR diagnosed by transesophageal echocardiography (TEE) that was successfully treated with immediate coronary artery bypass grafting (CABG).

## Case presentation

**Fig. 1:**
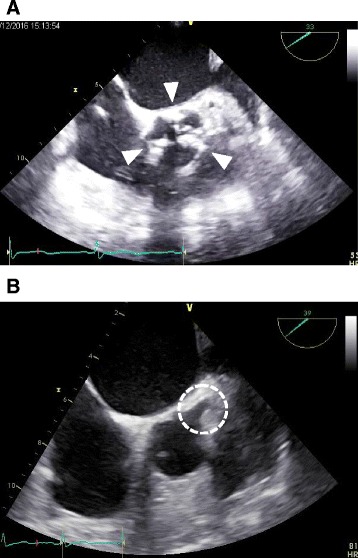
**a** Preoperative transesophageal echocardiography. Severe calcification was observed in all leaflets and aortic annulus (*arrowheads*). **b** Preoperative transesophageal echocardiography. The left coronary ostium was intact; there was no significant stenosis (*dotted circle*)

**Fig. 2:**
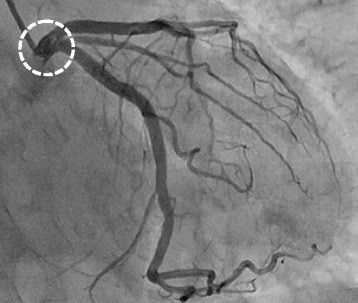
Preoperative coronary angiography. Left coronary ostium did not show significant stenosis (*dotted circle*)

An 80-year-old woman (height, 142 cm; weight, 48 kg) with severe aortic stenosis was scheduled to undergo AVR. A subjective symptom was exertional dyspnea. Exercise tolerance was 2METs. Preoperative transthoracic echocardiography (TTE) revealed the aortic valve to be tricuspid, with a valve area of 0.57 cm [2], maximum flow rate of 5.0 m/s, maximum pressure gradient of 101 mmHg, and average pressure gradient of 64 mmHg. Severe calcification was observed in all leaflets and aortic annulus. Left ventricular wall motion was normal, and the ejection fraction was 77.5% (modified Simpson’s method). As well as TTE, TEE showed severe calcification of the aortic leaflets and annulus (Fig. 1a). TEE revealed the left coronary ostium intact (Fig. 1b). Similarly, preoperative coronary angiography did not reveal significant stenosis (Fig. 2).

Anesthesia was induced with intravenous administration of midazolam, remifentanil, and rocuronium and was maintained with intravenous remifentanil and rocuronium and desflurane inhalation. Desflurane inhalation was stopped and intravenous propofol started just prior to establishment of cardiopulmonary bypass (CPB). Intraoperative TEE revealed severe calcification around all leaflets and the valve annulus, but left ventricular wall motion was normal. CPB was established by cannulating the ascending aorta and right atrium, and the ascending aorta was clamped following cannulation of the aortic root. Cardioplegic solution was administered via the cannula, followed by dissection of the aortic root upon asystole. During AVR, cardioplegic solution was administered from each coronary ostium via a selective coronary artery cannula. A biological valve (19-mm diameter; Trifecta™ Valve) was replaced after disconnecting the native valve and careful decalcification of the annulus. Withdrawal from CPB was uneventful and did not require vasopressors. TEE showed minor paravalvular leakage and good left ventricular function without dysfunction.

**Fig. 3:**
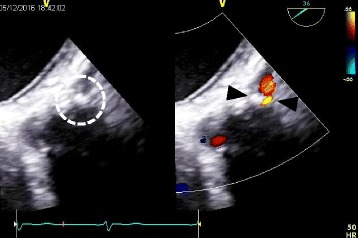
Intraoperative transesophageal echocardiography. *Left*, a high-brightness massive shadow at the left coronary ostium (*dotted circle*). *Right*, color flow on Doppler showed a mosaic image and accelerated blood flow (*arrowhead*)

About 20 min after withdrawing CPB, signs of left heart failure appeared; systolic blood pressure was <70 mmHg, and mean pulmonary pressure was >40 mmHg. Despite administration of dobutamine (5 μg/kg/min) and adrenaline (0.1 μg/kg/min), hemodynamics did not improve. TEE showed regional wall motion decreases of the left ventricular anterior and septal walls. The electrocardiogram showed wide QRS and extensive ST elevation especially in V4 and V5. There was a high-brightness massive shadow at the left coronary ostium (Fig. 3, left) where the color flow on Doppler showed a mosaic image (Fig. 3, right). The patient was diagnosed with acute left heart failure owing to decreased left coronary perfusion. CPB was immediately resumed, and CABG to the left anterior descending artery with the large saphenous vein was performed. After CABG, regional wall motion at anterior walls was still moderately hypokinetic but wall thickness was on improving trend. Motion of septal walls improved markedly and became normal with catecholamines. TEE showed remnant of high-brightness massive shadow at the left coronary ostium with no mobility, and the color flow on Doppler showed a mosaic echo remain.

**Fig. 4:**
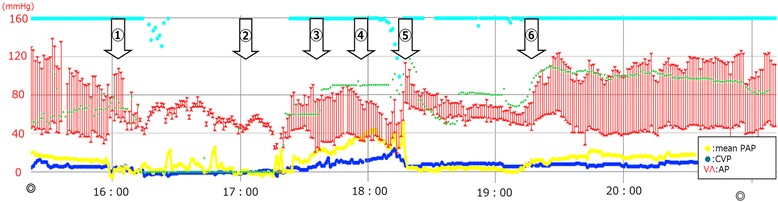
Anesthetic record. ◎ start/end of surgery. *PAP* pulmonary artery pressure, *CVP* central venous pressure, *AP* arterial pressure, ① start of CPB, ② AVR, ③ withdrawal of CPB, ④ signs of left heart failure and appearance of left ventricular wall motion abnormality, ⑤ second run of CPB, ⑥ second withdrawal of CPB

CPB was withdrawn with administration of dobutamine (5 μg/kg/min) and adrenaline (0.1 μg/kg/min). Hemodynamics was stabilized following CPB (Fig. 4).

Details of the operation were as follows: operative time, 331 min; duration of anesthesia, 403 min; duration of extracorporeal circulation, 177 min; duration of aortic interruption, 63 min; volume of infusion: blood transfusion (3300 ml), red blood cell solution (6 units), and fresh frozen plasma (10 units); urine volume, 475 ml.

## Discussion

Following the withdrawal of CPB and onset of acute left heart failure, TEE showed a high-brightness massive shadow at the left coronary ostium (Fig. 3) that was absent prior to surgery (Fig. 2). The massive shadow was considered part of the intimal piece that peeled off during decalcification and removal of calcified lesions in the valve annulus. We believe that as the cardiac output is increased, the floating intimal piece obstructs the coronary origin, leading to coronary ostial stenosis.

Coronary ostial stenosis is a fatal condition resulting from obstruction of the coronary ostia in the subacute phase following AVR (typically 1–6 months post-AVR). It can lead to, e.g., angina, left heart failure, and acute pulmonary edema. Thus, early diagnosis and treatment are crucial. Its frequency of onset is 1 to 5% and is diagnosed by coronary angiography and treated by coronary artery bypass surgery or percutaneous coronary angioplasty [1–7].

Although the causes of coronary ostial stenosis are not clear, various reports have suggested potential pathogenic mechanisms. For instance, Funada [1] reported three patients who developed coronary ostial stenosis 1 to 6 months after AVR with direct myocardial protection/perfusion. These patients exhibited a wide range of ostial fibrosis by intravascular echocardiography. In another study, Roberts and Morrow [8] reported postoperative pathological changes including fibrotic thickening of the endothelium of the coronary artery upon necropsy of patients after AVR. Based on these observations, direct cannulation of the coronary artery ostium and myocardial protection/perfusion appear to cause acute traumatic tissue damage and tissue remodeling during the trauma healing process, leading to delayed coronary ostial stenosis [1].

Coronary ostial stenosis is generally regarded as a late-onset disease, and only three cases [9–11] have been reported with perioperative onset. In two of the three cases, the prosthetic valve itself was the cause of stenosis. In one report by Umran [9], calcified lesions remaining in the right coronary ostium was thought to have caused a calcium embolism. In that study, right heart failure and right coronary ostial stenosis were diagnosed after AVR but before withdrawal of CPB. Thus, CABG was performed to the right coronary artery and an intra-aortic balloon pump (IABP) was inserted, followed by withdrawal of CPB.

The pathogenesis of coronary ostial stenosis in the present case is similar to that reported by Umran, although the timing of onset differs. In the present case, once CPB was withdrawn and circulation dynamics stabilized, left heart failure due to left coronary ostial stenosis occurred. Thus, CPB was established again and CABG was performed. As left heart function improved after CABG, it was possible to withdraw the patient from CPB without the need for an IABP.

Coronary ostial stenosis due to remnant intimal calcified lesions may occur at any time after CPB establishment and can be effectively diagnosed by TEE.

Regional wall motion abnormality of left ventricle and left heart failure due to coronary air embolism sometimes occur at heart surgery with CPB. It often occurs in early stage of withdrawal of CPB because of left heart chamber’s remnant air. Although the onset time is the same at this case, there are some different points. First, the risk of air embolism is considered to be low because remnant air in the heart chamber was vented carefully observing with TEE. Second, a brightness massive shadow at the left coronary ostium was brighter than the shadow of air and did not move like floating air. After CABG, TEE showed remnant of high-brightness massive shadow with no mobility. Finally, in general, air embolism occurs in the right coronary artery which is on the belly side; however, in this case, left coronary embolism happened. Considering the above things comprehensively, massive air embolism was considered negative.

## Conclusions

In the present case, acute left heart failure developed after CPB withdrawal during AVR. Left coronary ostial stenosis was diagnosed by TEE and treated with CABG.

## Abbreviations

AVR: Aortic valve replacement
CABG: Coronary artery bypass grafting
CPB: Cardiopulmonary bypass
IABP: Intra-aortic balloon pump
TEE: Transesophageal echocardiography
TTE: Transthoracic echocardiography
